# The postbiotic potential of *Aspergillus oryzae* – a narrative review

**DOI:** 10.3389/fmicb.2024.1452725

**Published:** 2024-10-23

**Authors:** Yvonne Seidler, Gerald Rimbach, Kai Lüersen, Gabriel Vinderola, Ignacio R. Ipharraguerre

**Affiliations:** ^1^Institute of Human Nutrition and Food Science, Division of Food Science, Faculty of Agricultural and Nutritional Sciences, University of Kiel, Kiel, Germany; ^2^Instituto de Lactología Industrial (CONICET-UNL), Faculty of Chemical Engineering, National University of Litoral, Santa Fe, Argentina

**Keywords:** *Aspergillus oryzae*, postbiotic, immune modulation, fungal metabolites, gut microbiota

## Abstract

The filamentous fungus *Aspergillus oryzae* has a long tradition in East Asian food processing. It is therefore not surprising that in recent years fermentation products of *A. oryzae* have attracted attention in the emerging field of postbiotics. This review aims to provide a comprehensive summary of the potential postbiotic effects of fermentation products from *A. oryzae*, by discussing possible mechanisms of action against the background of the molecular composition determined so far. In particular, cell wall constituents, enzymes, extracellular polymeric substances, and various metabolites found in *A. oryzae* fermentation preparations are described in detail. With reference to the generally assumed key targets of postbiotics, their putative beneficial bioactivities in modulating the microbiota, improving epithelial barrier function, influencing immune responses, metabolic reactions and signaling through the nervous system are assessed. Drawing on existing literature and case studies, we highlight *A. oryzae* as a promising source of postbiotics, particularly in the context of animal health and nutrition. Challenges and opportunities in quality control are also addressed, with a focus on the necessity for standardized methods to fully harness the potential of fungal-based postbiotics. Overall, this article sheds light on the emerging field of *A. oryzae*-derived postbiotics and emphasizes the need for further research to fully realize their therapeutic potential.

## 1 Introduction

Fungi, with their exceptional diversity and versatility, play pivotal roles in ecological (Coleine et al., 2022; Bahram and Netherway, 2022), industrial (Sharma and Rath, 2021; Kumar et al., 2023; Arnau et al., 2020), and biomedical domains (Bachheti et al., 2021; Gupta et al., 2020; Hashem et al., 2023). As natural decomposers, they are fundamental to nutrient cycling within ecosystems, by breaking down complex organic materials into simpler monomeric forms (Kong et al., 2022; Lustenhouwer et al., 2020; Fukasawa and Matsukura, 2021). This enzymatic process, facilitated by a robust arsenal of different hydrolases not only supports ecosystem functioning but also enables the production of commercially valuable substances like ethanol (Nogueira et al., 2020; Tse et al., 2021) and organic acids (Dusengemungu et al., 2021; Andrino et al., 2021). Additionally, fungi enhance various processes in the food industry such as fermentation which is vital for the production of bread (Dahiya et al., 2020), cheese and meat (Ropars and Giraud, 2022), and beverages (Takeshita and Oda, 2023). In the medical field, fungi provide potent pharmaceuticals like antibiotics and are investigated for their ability to produce diverse secondary metabolites with therapeutic properties, including statins and other bioactive compounds (Devi et al., 2020; Adeleke and Babalola, 2021; Ancheeva et al., 2020). While historically recognized for their nutritional and medicinal value (Nagaoka and Hara, 2019; Pandey et al., 2019; Fleming, 1929; Wasser, 2002), fungi also produce mycotoxins such as aflatoxins, ochratoxin A, and fumonisins, which pose significant health risk (Perrone and Gallo, 2017).

*Aspergillus oryzae*, commonly known as “Koji mold,” plays a crucial role in the fermentation of various food products in East Asian cuisine (Liu et al., 2024; Yamashita, 2021). The *A. oryzae*-based preparation of koji, which has a long tradition of more than a 1000 years (Kusumoto et al., 2021), is used in the production of sake (rice wine), shoyu (soy sauce), amazake (rice koji beverage), osu (rice vinegar), kurosu (black rice vinegar), shochu (distilled alcoholic beverage fermented with koji), and miso (soybean paste) (Kusumoto et al., 2021; Ichishima, 2022; Murooka, 2020; Takeshita and Oda, 2023; Gall and Benkeblia, 2023). To start the fermentation process, the fungus is added to a substrate such as rice or soybeans, where it grows and produces enzymes that break down the starches, proteins, and fats of the substrate into simpler compounds including sugars, amino acids, and fatty acids. These compounds are then further fermented by other microorganisms, such as yeast and lactic acid bacteria, to generate the final food products (Liu et al., 2024). The beneficial involvement of koji molds in Japanese culture has propelled research and advancements in areas such as academia, industry, medicine, and agriculture (Yamashita, 2021; Daba et al., 2021; Kitagaki, 2021).

**Figure 1:**
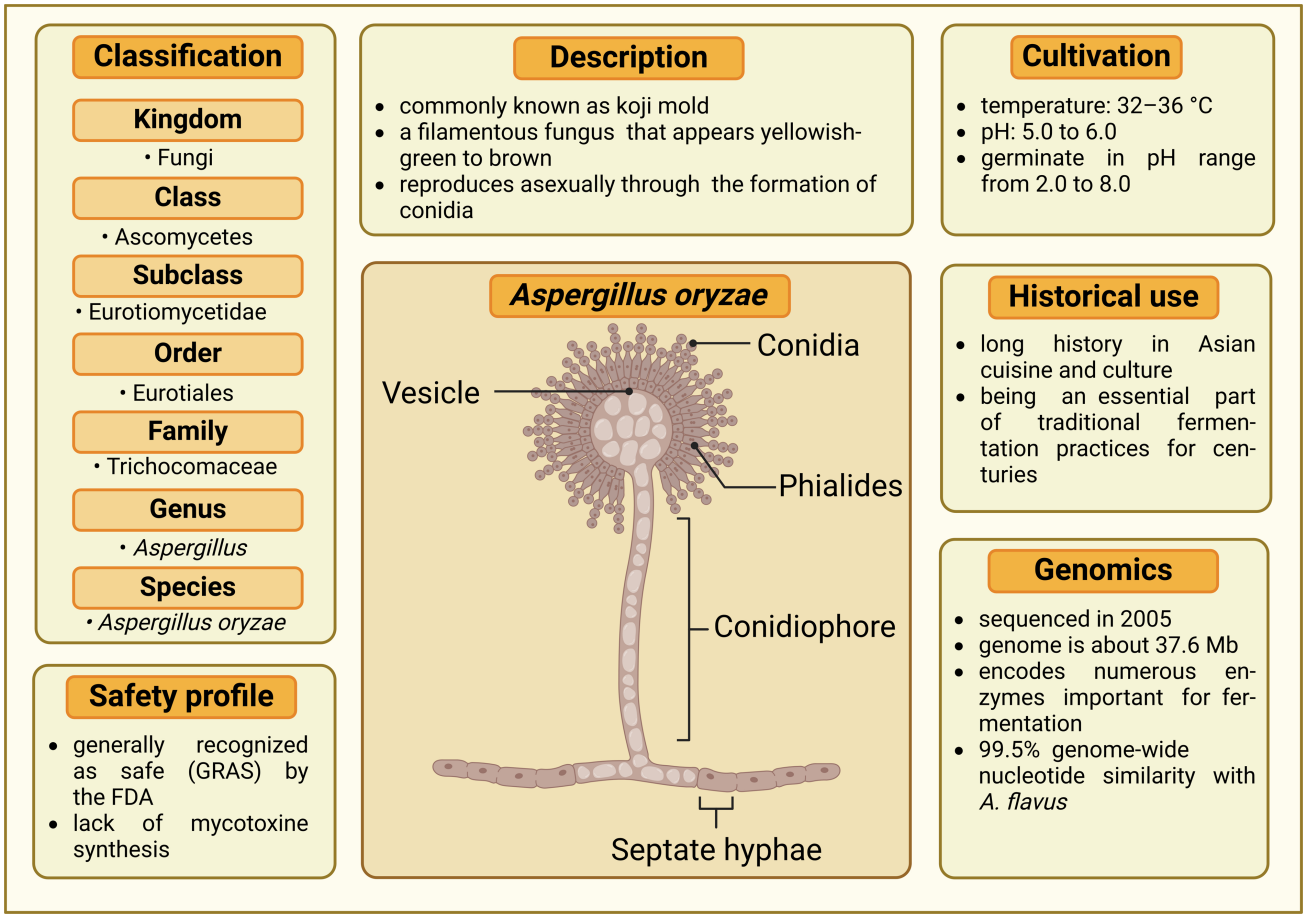
Comprehensive profile of *Aspergillus oryzae*. The central illustration depicts the morphological structures of *A. oryzae*, including vesicle, conidia, phialides, conidiophore, and septate hyphae. Surrounding the illustration are key aspects of the fungus, covering its classification, safety profile, description, cultivation requirements, historical use, and genomic characteristics. Created in BioRender. Seidler, Y. (2024) BioRender.com/i28s583.

*A. oryzae* belongs to a genus of filamentous fungi (its taxonomic classification is depicted Figure 1) that includes a variety of species, some of which are beneficial for human use, while others are pathogenic or produce toxic compounds. *Aspergillus* molds often reproduce through both sexual (Asgarivessal et al., 2023) and asexual methods (Ojeda-López et al., 2018), although some species, including *A. oryzae*, are primarily recognized for reproducing exclusively asexually (Wada et al., 2012). The structure of the conidiophore (see the morphology of *Aspergillus* in Figure 1), which carries the asexual spores, is a crucial characteristic used for taxonomic classification in *Aspergillus* (Suleiman, 2023). Among the approximately 400 *Aspergillus* species (Ibrahim et al., 2023), the most widely recognized members are *A. flavus* and *A. parasiticus*, which are mycotoxin producers (Ting et al., 2020; Priesterjahn et al., 2020). Their infestation of crops like maize, peanuts, and beans, coupled with the production of highly carcinogenic and mutagenic mycotoxins, notably aflatoxin B1, poses significant health threats to both humans and animals (Fouad et al., 2019). *A. fumigatus* is another significant species, predominantly known as a pathogen that can cause invasive aspergillosis, particularly in immunocompromised individuals (Latgé and Chamilos, 2019). The beneficial *Aspergillus* species *A. oryzae* and the closely related *A. sojae* are regarded by most taxonomists as the domesticated forms of *A. flavus* and are widely used in food industry (Frisvad et al., 2018; Daba et al., 2021; Frisvad et al., 2019). Typically, *A. oryzae* thrives best at temperatures between 32 and 36°C and is unable to grow beyond 44°C. It prefers a pH range of 5.0 to 6.0 for growth, but can germinate in conditions with pH levels from 2.0 to 8.0 (Daba et al., 2021).

The genome of *A. oryzae* has a size of 37.6 Mb (Machida et al., 2005), which is 20–30% larger than the genomes of other *Aspergillus* species such as *A. nidulans* and *A. fumigatus* (Nierman et al., 2005; Galagan et al., 2005). This difference in genome size is attributed to the presence of a higher number of transposable elements and gene duplications in *A. oryzae*. Remarkably, despite the larger genome of *A. oryzae*, there are almost no genotypic differences between *A. oryzae* and *A. flavus*, with the two species sharing 99.5% genome-wide nucleotide similarity (Han et al., 2024). The *A. oryzae* genome contains approximately 12,000 genes distributed on eight chromosomes that encode a wide variety of enzymes, including amylases, proteases, and lipases, which contribute to the fungus’s ability to degrade complex organic materials and are essential for its use in fermentation processes (Machida et al., 2005). While the genetic structure of numerous *A. oryzae* strains has been decoded (Machida et al., 2005; Zhong et al., 2018; Deng et al., 2018; Umemura et al., 2012; Zhao et al., 2012) functional genomics is still in its nascent stage in terms of developing strains for industrial purposes. This is evident from the fact that so far only around 200 genes have been functionally verified, making up a minimal of 1.7% of the whole genome (He et al., 2019).

*A. oryzae* is commonly classified as a fungus that is not pathogenic (Kitagaki, 2021). Moreover, it has not been associated with any carcinogenic compounds, including aflatoxins (Barbesgaard et al., 1992). This has been confirmed for different koji-mold strains of *A. oryzae*, which were all tested negative for aflatoxins (Tanaka et al., 2006). While there are *Aspergillus* strains that can produce certain types of mycotoxins (Kato et al., 2011), the fermentation industry consistently verifies that the levels of mycotoxins in their products are within the limits set by health authorities. Interestingly, *A. oryzae* has also been shown to degrade aflatoxins. A product called “D-Tox,” developed from *A. oryzae*, can reduce aflatoxin B1 (AFB1) by up to 90%, offering a promising approach to improving food safety (Choi et al., 2024).

Although *A. oryzae* and *A. flavus* are genotypically very similar (Han et al., 2024), *A. oryzae* is used in food production and listed as “Generally Recognized as Safe” (GRAS) status by the Food and Drug Administration (FDA) and has been approved as a safe microorganism by the World Health Organisation (WHO) (He et al., 2018a), whereas *A. flavus* is harmful for human, plants, and animals (Fouad et al., 2019; Amaike and Keller, 2011). It is suspected that there are disabling mutations in the gene cluster, which result in *A. oryzae* not producing aflatoxins, whereas *A. flavus* does (Tominaga et al., 2006; Tao and Chung, 2014). Subsequent studies, particularly at the molecular level, reinforced these findings. The aflatoxin production mechanism in *A. flavus* involves a series of over 25 genes. Through polymerase chain reaction (PCR) evaluations, it was observed that 15 out of 39 *A. oryzae* strains had missing sequences in five genes that align with the *A. flavus* aflatoxin gene cluster. In the other strains analyzed, the genes in the corresponding cluster were found to be non-functional (Kusumoto et al., 2000). Chang (2019) compared 13 *A. flavus* and 11 *A. oryzae* genome sequences based on genome-wide total single nucleotide polymorphism (total SNPs). The study proved a new technique to distinguish between *A. flavus* and *A. oryzae*.

No cases of invasive growth or systemic infections caused by *A. oryzae* have been reported in healthy individuals. However, there have been rare instances where strains identified as *A. oryzae* were isolated from individuals with compromised health, suggesting that while *A. oryzae* possesses a low potential for pathogenicity, it can grow in human tissue under extraordinary conditions, similar to many other detrimental microorganisms (Stelmaska et al., 1974; Liao et al., 1988; Tomizawa, 1981). There have been a limited number of reported cases of allergic reactions primarily attributed to *A. oryzae*, but these cases likely involved individuals already prone to allergic reactions and who were exposed to a significant amount of conidia via inhalation (Kino et al., 1982; Barbesgaard et al., 1992). Taken together, the demonstrated safety of *A. oryzae* qualifies it as a favored progenitor organism for not only the fermentation of foods but also the synthesis of a wide array of enzymes and chemicals with prospective medicinal benefits that could be harnessed for forthcoming medical procedures (Yu et al., 2004).

In the realm of microbiology and food science, *A. oryzae* represents a subject of study, bridging ancient culinary practices with modern biotechnological applications. Esteemed for its pivotal role in the fermentation of a vast array of East Asian foods (Daba et al., 2021), *A. oryzae* is not merely a workhorse of traditional fermentation but also of interest in the burgeoning field of postbiotics. For example, recent investigations into its postbiotic potential reveal promising health benefits in animal models (Kaufman et al., 2021; Ríus et al., 2022), suggesting a broader applicability of *A. oryzae*-derived products in promoting well-being.

In this review, we explore the various constituents, molecules, and cell wall components of *A. oryzae*, including enzymes and both primary and secondary metabolites. Additionally, we introduce the concept of postbiotics and link the metabolites, cell wall components, and enzymes of *A. oryzae* with the five proposed modes of action of postbiotics (Salminen et al., 2021). These modes of action are crucial in understanding how compounds from *A. oryzae* can potentially confer benefits to the host, which is a defining feature of postbiotics. Each metabolite’s role is examined in the context of these bioactive mechanisms, helping to clarify their potential health benefits. Thus, this work not only compiles existing research on *A. oryzae* but also identifies knowledge gaps and suggests directions for future studies. Finally, we focus on the emerging challenges and opportunities in the quality control of postbiotics derived from *A. oryzae*, a novel scientific topic that has not been extensively discussed in the literature. This review aims to set a foundation for advancing the understanding and development of *A. oryzae* as a postbiotic.

## 2 Methodology

The content synthesized in this narrative review is derived from an exhaustive literature search employing various scholarly databases and scientific websites, including Scopus, Web of Science, PubMed, Google Scholar, and Science Direct. This search utilized a comprehensive set of keywords to capture the broad spectrum of research concerning *A. oryzae* including the term “*Aspergillus oryzae*” in combination with the term’s “enzymes,” “compounds,” “fermentation,” “primary compounds,” “secondary compounds,” “secondary metabolism,” “cell wall,” “biological activity,” “prebiotic,” “probiotic,” “postbiotic,” “heat inactivated,” “inactivated,” “killed,” “dried,” “inanimated,” and “non-viable,” respectively.

Additionally, the selection process prioritized peer-reviewed articles, reviews, and significant research reports, while conference papers and abstracts were excluded unless they provided novel insights or data unavailable elsewhere. The search strategy included scanning titles, abstracts, and full-texts to ensure relevance to *A. oryzae’s* diverse roles and applications in enzymatic processes, fermentation, and bioactivity.

Each keyword was carefully selected to ensure that all relevant aspects of *A. oryzae*, including its primary and secondary metabolites, and its use as a biotic agent, were thoroughly explored. Furthermore, we ensured that studies addressing safety, industrial applications, and advances in biotic and postbiotic research were also covered, thereby providing a holistic overview of the literature.

## 3 Fermentation of *Aspergillus oryzae*

### 3.1 Solid state and submerged fermentation

In general, fermentation is defined as a biochemical process in which microorganisms such as bacteria and fungi break down complex compounds into simpler substances and generate energy under anaerobic conditions (Nout, 2005). Yet, fermentation in a biotechnical context can be aerobe (culturing) and anaerobe as for instance in soy sauce production (Ito and Matsuyama, 2021). During this process, microorganisms produce primary and secondary metabolites. These bioactive compounds, which include antibiotics, peptides, enzymes, and growth factors, have significant industrial and economic value (Robinson et al., 2001; Balakrishnan, 1996). As the demand for these compounds has grown, techniques have been scaled up from laboratory settings to industrial levels. This scaling presents challenges in maintaining controlled environments for microbial growth, as deviations can lead to undesired (by-)products (Subramaniyam and Vimala, 2012). Depending on the nature of the substrate, solid state fermentation (SSF) is distinguished from submerged fermentation (SmF). The former is characterized as a fermentation procedure where microorganisms grow on solid substrates in the absence of free-flowing liquid (Wang J. et al., 2023). One of the primary advantages of utilizing solid substrates is the opportunity to efficiently recycle nutrient-rich waste (El Sheikha and Ray, 2023; Paz-Arteaga et al., 2023). In this approach, substrates undergo a slow and consistent consumption, facilitating prolonged fermentation periods and a controlled release of nutrients. SSF is especially advantageous for fungi and certain microorganisms that prefer environments with reduced moisture content. On the other hand, SmF is a sophisticated fermentation technique utilizing liquid media and substrates, including molasses and various broths. This method facilitates the secretion of bioactive compounds directly into the fermentation broth (Xv et al., 2024). SmF offers precise control over essential parameters, such as temperature, aeration, and agitation, all pivotal for maximizing product yield. The meticulous regulation of these factors, especially temperature, impacts the growth rate, oxygen dynamics, and overall product synthesis of the microorganism (Esmeralda-Guzmán et al., 2024). The choice between SSF and SmF often depends on the specific bioactive compound being produced and the substrate used in the fermentation process (Premalatha et al., 2023; Sankar et al., 2023).

As far as *A. oryzae* is concerned, the production of various foods through SSF (Rousta et al., 2021) on diverse substrates such as rice, wheat bran, and soybeans has a long tradition. Moreover, SmF with *A. oryzae* is frequently employed for the production of enzymes (Masuda et al., 2009; Shah et al., 2014) and organic acids (Singh et al., 2022; Badar et al., 2021). Both fermentation techniques have been developed for large-scale production.

### 3.2 Stress response and physiological adaptation during fermentation

During fermentation, the microorganism used has to adapt to various abiotic factors including temperature, pH, oxidative, and osmotic conditions, which can sometimes cause stress (Hagiwara et al., 2015). In the context of soy sauce fermentation, for instance, *A. oryzae* contends with elevated salt concentrations (17–18%) and an acidic pH (He et al., 2018b). The high salt concentrations induces stress due to the disruption of osmotic potential (Taymaz-Nikerel et al., 2016). Research by He et al. (2018b) highlighted that salt stress led to changes both at the transcriptome and metabolome levels in *A. oryzae* with upregulated expression of genes related to arginine accumulation and oleic acid synthesis. Additionally, variations in lipid metabolism in response to salt stress were observed, notably leading to an increase in intracellular linoleic acid. Recent studies further show that reducing salt during soy sauce production enhances *A. oryzae’s* carbon and protein metabolism, which not only improves substrate efficiency but also supports *Lactobacillus* growth and balances the microecology. This adaptation also boosts the production of smoky, nutty, and malty aromas in soy sauce while reducing rancid odors by suppressing fatty aldehyde production through enhanced *β*-oxidation (Liu et al., 2024).

During rice wine fermentation, *A. oryzae* undergoes ethanol-induced stress. Ma et al. (2019) elucidated that ethanol absorption led to cellular perturbations and a rise in fatty acid unsaturation. This was evidenced by the conversion of stearic acid to linoleic acid and the heightened expression of related fatty acid desaturases. Temperature fluctuations present another challenge for *A. oryzae* during fermentation. Both high and low temperatures can hinder the growth and conidial formation. On a molecular level, temperature changes were reported to influence the expression of genes associated with sugar and lipid metabolism. Specifically, low temperatures stimulated genes related to trehalose synthesis and starch metabolism, while high temperatures suppressed genes involved in the metabolism of fructose, galactose, and glucose (Jiang et al., 2022). Furthermore, in the context of glucosamine (GlcN) production from *A. oryzae* NCH-42, environmental factors such as nitrogen sources, temperature, and pH play a pivotal role in determining the cell wall composition. Li W. et al., 2021 found that acidic stress, particularly at a pH of 2.5, significantly enhanced GlcN production, with the fungal biomass yielding up to 0.31 g/g of GlcN. This was corroborated by scanning electron microscopy (SEM) examinations that revealed a robust mycelial structure under these conditions (Li J. S. et al., 2021).

The research on *A. oryzae* demonstrates that a variety of abiotic factors, along with the type of fermentation and the substrate used, influence the chemical composition of *A. oryzae* fermentation end products. Consequently, it can be inferred that depending on the actual fermentation conditions end products from the same species may vary considerably in their effectiveness to cause beneficial effects and may not necessarily operate through the same mechanisms.

## 4 Postbiotics – an emerging concept in the field of “biotics”

### 4.1 From prebiotics and probiotics to postbiotics

Prebiotics were originally defined as “a non-digestible food ingredient that beneficially affects the host by selectively stimulating the growth and/or activity of one or a limited number of bacteria already resident in the colon” (Gibson and Roberfroid, 1995). This concept has its roots in early research, such as the study by Rettger and Cheplin (1921) who observed the enrichment of human microbiota with lactobacilli after carbohydrate consumption. Over time, the definition of prebiotics has evolved. The most recent version, provided by the International Scientific Association for Probiotics and Prebiotics (ISAPP), defines a prebiotic as “a substrate that is selectively utilized by host microorganisms conferring a health benefit” (Dubos et al., 1965; Savage, 1977). Common examples of prebiotics include inulin, fructooligosaccharides (FOS), and galactooligosaccharides (GOS), which are preferentially metabolized by bifidobacterial (Gibson et al., 2017).

In contrast, probiotics—first introduced by Lilly and Stillwell (1965)—refer to live microorganisms. In, 2001, the WHO and FAO defined probiotics as “live microorganisms which, when administered in adequate amounts, confer a health benefit on the host” (FAO/WHO, 2001). Synbiotics refer to a combination of probiotics and prebiotics that either function independently or synergistically to provide health benefits (Markowiak and Śliżewska, 2018; Swanson et al., 2020).

As research has advanced, additional terms such as paraprobiotics (non-active probiotic cells) and postbiotics have been introduced, emphasizing that non-living cells, whether whole or fragmented, can also positively affect human health. The ISAPP defines postbiotics as “a preparation of inanimate microorganisms and/or their components that confers a health benefit on the host” (Salminen et al., 2021). The core of the definition are inanimate microbes, metabolites can be present, or not, in the final product. Metabolites alone, in the absence of inanimate cells, are not regarded as postbiotics according to the definition. Common misunderstanding around the definition of postbiotics was recently clarified (Vinderola et al., 2024).

**Figure 2:**
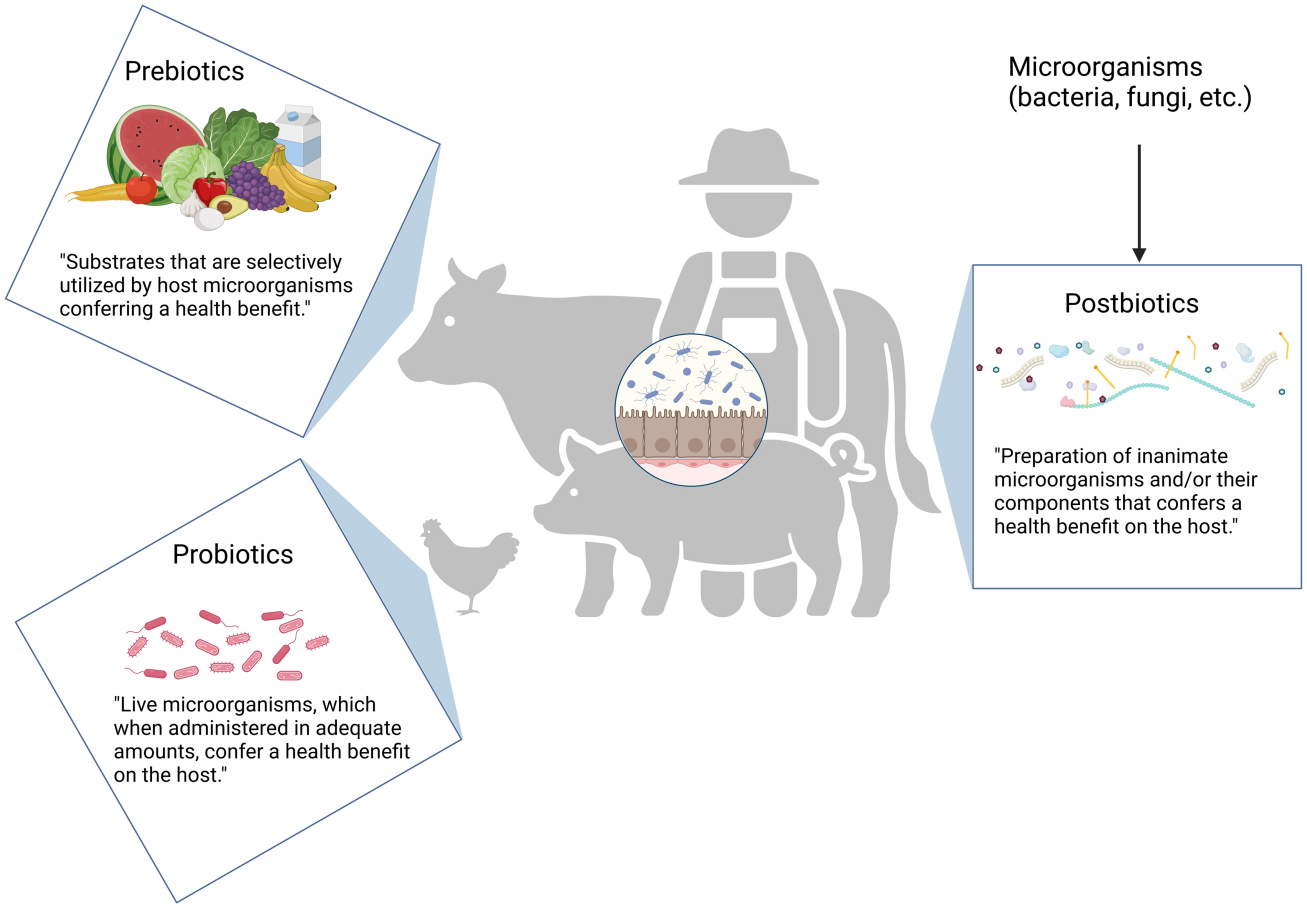
Definition of prebiotics, probiotics, and postbiotics. Prebiotics are specific dietary compounds that beneficially nurture the host’s existing microorganisms. They do not contain live organisms but fuel beneficial bacteria already present in the gut. Examples include soluble fibers such as inulin, fructooligosaccharides, and galactooligosaccharides. Probiotics refer to live microorganisms that, when consumed in adequate amounts, confer health benefits on the host. These are the “good” bacteria introduced into the system to enhance gut health and other functions. Only those strains with scientifically demonstrated health effects are termed as “probiotic-like.” Postbiotics, as the name suggests, are derived “after life.” They may include substances obtained from microbial activity once the microorganisms are no longer alive. This category can encompass a range of components, from cell wall fragments and enzymes to amino acids, organic acids, and various metabolites. Created in BioRender. Seidler, Y. (2024) BioRender.com/h54l897.

Besides inanimate microorganisms, a postbiotic product may include metabolites generated by microorganisms, such as short chain fatty acids (SCFA), exopolysaccharides, cell wall fragments, enzymes/proteins, cell-free supernatant, and other metabolites (Żółkiewicz et al., 2020). In a 2021 publication by ISAPP, various proposed mechanisms of postbiotics were outlined. Accordingly, postbiotics are suggested to potentially modulate gut microbiota dynamics, booster intestinal barrier integrity, exhibit immunomodulatory effects, regulate systemic metabolism, and/or engage in signaling through the nervous system. A comprehensive description of these mechanisms can be found in the publication by Salminen et al. (2021). The differences between prebiotics, probiotics, and postbiotics are illustrated in Figure 2.

## 5 Possible bioactive compounds in *Aspergillus oryzae* fermentation end products

According to the definition proposed by Salminen et al. (2021), fermentation end products from *A. oryzae* fermentation can be categorized as postbiotics as long as they do not contain living cells. Given that postbiotics can include a combination of non-viable cells, cell fragments and metabolites of the progenitor microorganism, it is expected that their components bearing bioactivity can exhibit considerable diversity. This diversity will be examined in the subsequent section, with a primary emphasis on compounds derived from *A. oryzae*. However, the specific bioactivities and health benefits associated with these compounds will be addressed in detail in section 7, in order to avoid redundancy and ensure clarity.

### 5.1 Cell wall fragments of *Aspergillus* species

The cell walls of filamentous fungi are complex structures composed mainly of polysaccharides (90%) (Latgé, 2010), which differ significantly from the cellulose-based plant cell walls (Bowman and Free, 2006). The fungal cell wall has been simplistically separated into a fibrillar alkali-insoluble skeleton and an amorphous alkali-soluble cement (Latgé, 2010). At present, there is no detailed description of the cell wall of *A. oryzae*. For this reason, we have mainly reviewed evidence from *A. fumigatus*, which cell wall has been dissected in depth at the molecular level. However, it is important to note that while cell wall components of *A. fumigatus* are implicated in causing 50–90% of human aspergillosis cases (Latgé and Chamilos, 2019), *A. oryzae* is a non-pathogenic species considered as safe. Even though we generalize between the two *Aspergillus* species in this review due to their taxonomic proximity, the reader should be aware that biologically relevant differences in their cell wall composition and/or structure likely exist.

**Figure 3:**
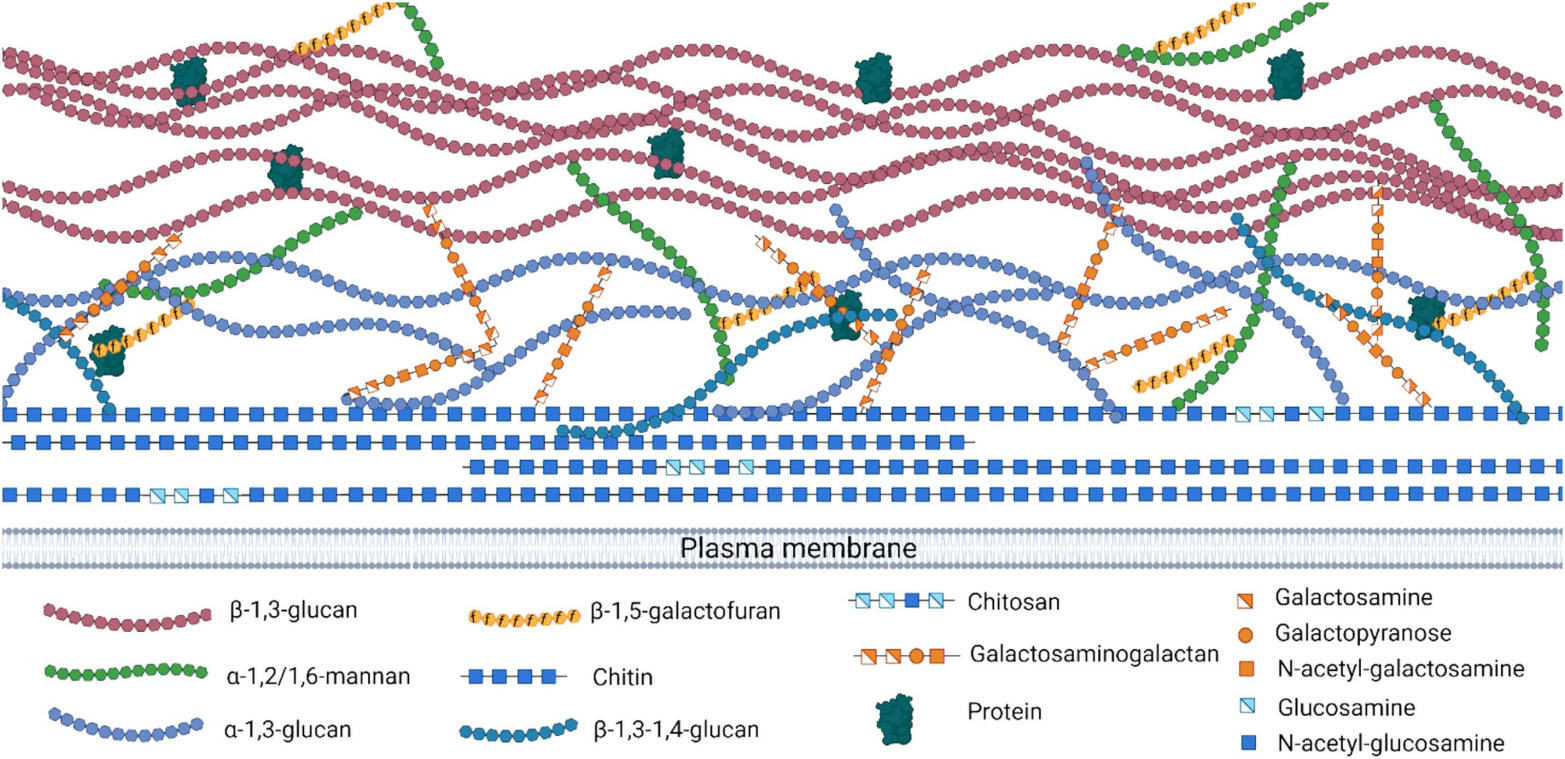
*Aspergillus* spp. cell wall organization and polysaccharides. Structural depiction of the cell wall polysaccharides showing linkages and monosaccharide components. Created with Biorender.com.

The main polysaccharides in the *Aspergillus* cell wall are *α*-glucans (α-1,3-glucan and α-1,4 glucan), *β*-glucans (β-1,6-branched β-1,3-glucan and linear β-1,3/1,4-glucan), galactosaminogalactan (GAG), galactomannan (GM), and chitin (linked via β-1,4 linkage to β-1,3-glucan) (Figure 3). These polysaccharides have been characterized in detail in *A. fumigatus* (Fontaine et al., 2000; Mouyna and Fontaine, 2008).

The polysaccharide α-1,3-glucan stands as a hallmark component of the outer cell wall in *A. fumigatus* (Lee and Sheppard 2016). Comprised of glucose units interconnected through α-1,3 linkages, its presence, while not unique to *A. fumigatus*, holds relevance in the context of bioactivity-bearing compounds. In the case of *A. oryzae*, the production of α-1,3-glucan is orchestrated by three synthases: Ags1, Ags2, and Ags3 (Miyazawa et al., 2016; Zhang et al., 2017). In studies focusing on *A. oryzae*, *ags* gene knockouts have led to enhanced recombinant enzyme production (Miyazawa et al., 2016), and it was observed that α-1,3-glucan acts as a potential inhibitory factor for enzymes such as α-amylase (Zhang et al., 2017). Additionally, the deletion of α-1,4-glucan in *A. oryzae* results in an increased accumulation of α-1,3 glucan (Koizumi et al., 2023).

β-1,3-glucan is a primary structural component of the *A. fumigatus* hyphal cell wall, consisting of glucose residues connected by *β*-1,3 linkages (Lee and Sheppard 2016). In a comparative study involving *Saccharomyces cerevisiae*, *Xanthomonas campestris*, and *Bacillus natto*, *A. oryzae* stood out by producing the highest mass of β-glucan during SmF conditions (Utama et al., 2021). Additionally, SSF with *A. oryzae* was shown to increase the β-glucan content of brown rice used as a substrate (Ji and Ra, 2021).

In *A. fumigatus,* the inner cell wall structural integrity is mainly attributed to the presence of chitin, a pivotal structural polysaccharide. Chitin, consisting of N-acetyl-glucosamine (GlcNAc) residues linked by β-1,4 bonds, is a fundamental component of the fungal cell wall carbohydrate structure, though its prevalence differs among fungal species (Lenardon et al., 2010; Munro and Gow, 2001; Latgé, 2007). For instance, while it constitutes a mere 1–2% of the cell wall in *S. cerevisiae*, in filamentous fungi, it can represent up to 10–20% of the mycelial dry weight (Bartnicki-Garcia, 1968).

In *A. oryzae*, chitin significantly influences the structural integrity of the cell wall. Recent research has advanced the understanding of chitin’s biosynthesis in *A. oryzae* through the characterization of chitin synthase genes. The novel gene *chsZ* suggests a unique role in chitin synthesis, highlighted by its classification into a newly proposed class VI of chitin synthases (Chigira et al., 2002). Moreover, studies on the disruption of chitin synthase genes like *chsB* and *csmA* have shown that these alterations affect fungal morphology and growth dynamics without impacting *α*-amylase productivity, which influences the rheological properties of the cultivation broth in industrial fermentations (Müller et al., 2002a; Müller et al., 2002b).

Adjacent to chitin is its derivative, chitosan, which also composes the cell walls of numerous fungi. This compound emerges from the deacetylation of chitin, a process mediated by chitin deacetylases (CDA), culminating in the formation of glucosamine residues. This was described for the first time by Kreger (1954). Since then, two putative CDA-encoding genes have been identified in *A. fumigatus*, but their precise roles and contributions remain enigmatic (Gastebois et al., 2009). Chitosan’s physicochemical properties, notably its solubility and rigidity, are influenced by its degree of deacetylation (Kurita, 2006; El Knidri et al., 2018; Dash et al., 2011), which is defined as the molar fraction of deacetylated units in the polymer chain (Zhang et al., 2005). This has propelled chitosan to the forefront of various industrial applications, with notable inroads in the medical sector, particularly for the prevention of biofilm formation on medical devices (No et al., 2007; Salamanca et al., 2006; Boateng et al., 2008). A study investigated chitosan production by novel *A. oryzae* isolates grown under SmF conditions. One isolate (A2) identified by a 99% genetic similarity to known *A. oryzae* genome sequences, yielded the highest level of chitosan at 352 mg/L and a biomass of 9.48 g/L. Structural validation revealed a 55.23% degree of deacetylation for the chitosan, which displayed antimicrobial activity against several pathogens, most effectively against *Salmonella typhimurium* (Jebur et al., 2019).

GM is an immunoreactive substance (Latgé et al., 1994) widely distributed among most *Aspergillus* species (Barreto-Berter et al., 1980; Bardalaye and Nordin, 1977; Bennett et al., 1985; Reiss and Lehmann, 1979). In *A. fumigatus*, its complex structure is made up of mannose and galactofuranose (Gal*f*). A mannan chain serves as the backbone, with mannose residues connected via *α*-1,3 or α-1,6 linkages, while the side chains of the galactomannan consist of an average of 4 to 5 *β*-1,5-Gal*f* units (Latgé et al., 1994). The exact role of GM in virulence is still a topic of debate. While the mannan components, which are vital for maintaining cell wall structure, are non-antigenic, the role of the Gal*f* side chain in virulence remains not clearly defined (Lee and Sheppard, 2016; Lamarre et al., 2009; Schmalhorst et al., 2008). The absence of Gal*f* in *A. fumigatus* resulted in attenuated virulence (Schmalhorst et al., 2008) and GM detection using a monoclonal antibody that specifically reacts with Gal*f*-containing glycostructures serves as a diagnostic marker for *Aspergillus* infection, which emphasize the importance of understanding its synthesis and regulation (Heesemann et al., 2011; Marino et al., 2017). Nakajima and Ichishima (1994) isolated a GM-protein complex from *A. oryzae’s* hyphal walls, composed of 89% carbohydrates and 11% proteins, featuring a mannan backbone with predominantly β-Gal*f*-capped mannose side chains. Structural changes induced by chemical treatments indicated a complex architecture involving both *N*- and *O*-linked carbohydrate chains. Another study explored the biosynthesis and functional role of D-Gal*f*-containing glycans in *A. oryzae*. The deletion of the *ugmA* gene, encoding uridine diphosphate (UDP)-galactopyranose mutase, significantly impaired mycelial elongation, underscoring the importance of these glycans in maintaining cell wall integrity. Nuclear magnetic resonance analysis of the Δ*ugmA* mutant confirmed the presence of core mannan backbones, despite the absence of Gal*f*-containing sugar chains (Kadooka et al., 2023).

GAG is a complex sugar molecule present within the extracellular matrix as well as the inner and outer layers of the cell walls in *A. fumigatus* hyphae (Lee and Sheppard, 2016). This compound, made up of galactose and GalNAc connected through *α*-1,4 bonds, has a diverse structure due to the non-uniform positioning of its galactose and GalNAc units (Gravelat et al., 2013; Fontaine et al., 2011). The heterogeneity is unique to the GAG because most of the cell wall polysaccharides are homopolymers (chitin, glucans). Building upon the structural diversity of GAG in *Aspergillus* species, recent studies have elucidated its functional role in *A. oryzae*, particularly in mediating hyphal aggregation, which significantly impacts industrial fermentation processes. Research identified that GAG, alongside α-1,3-glucan, contributes to hyphal pellet formation. This was demonstrated by disrupting GAG biosynthesis in α-1,3-glucan-deficient mutants (AGΔ), resulting in the AGΔ-GAGΔ double mutant with fully dispersed hyphae in liquid culture, confirming GAG’s essential role in aggregation (Miyazawa et al., 2019). Further experimental addition of a partially purified GAG fraction to AGΔ-GAGΔ cultures induced mycelial pellet formation, highlighting GAG’s aggregation mechanism through acetylated galactosamine-mediated hydrogen bonding. This property not only affects hyphal structure but also enhances bioreactor production efficiency (Ichikawa et al., 2022). Additionally, *A. oryzae* NCH-42 was studied as a non-animal source of glucosamine (GlcN). Environmental factors like pH were examined, revealing that acidic conditions (pH 2.5) significantly boosted GlcN content (Li J. S. et al., 2021).

While sphingolipids are typically associated with the cell membrane rather than the structural framework of the cell wall, their inclusion in this context is warranted due to their relevance to *A. oryzae*. Investigations have revealed the presence and functional significance of glycosylceramides, shedding light on their potential health-promoting effects. For instance, Tani et al. (2014) identified *β*-glucosylceramide and β-galactosylceramide in *A. oryzae*. Similarly, Miyagawa et al. (2019) explored the cosmetic implications of glycosylceramides derived from various sources, including *A. oryzae*, on gene expression in human keratinocytes. These findings suggest that the abundance of glycosylceramides in *A. oryzae* may contribute to ceramide biosynthesis and tight junction formation in the skin, potentially elucidating the cosmetic benefits associated with koji. Additionally, Hamajima et al. (2016) investigated the prebiotic effects of koji glycosylceramide in murine models, unveiling its capacity to stimulate the proliferation of beneficial gut microbiota such as *Blautia coccoides*. The author suggest that the collective findings hint at a plausible nexus between Japanese dietary practices, gut microbiome modulation, and longevity via the consumption of koji-derived glycosylceramides.

### 5.2 Extracellular polymeric substances

Filamentous hyphae of *Aspergillus* grow embedded within an extracellular polymeric substance (EPS). In 1999, EPS were defined as all polymers outside the cell wall, which are not directly anchored to the outer membrane or murein-protein layer (Nielsen and Jahn, 1999). This extracellular matrix mediated adherence to inorganic substances and host cells and enhanced resistance to host defense and antifungal agents. The matrix is composed of heterogeneous macromolecules of extracellular DNA, proteins, lipids, and polyols, and exopolysaccharides including α-glucans, GM, and GAG, being carbohydrates and proteins usually the major components of EPS (Sheng et al., 2010). In *A. fumigatus*, the exopolysaccharides GAG, GM, α- and β-glucans are found, whereby GAG plays a critical role in the maintenance of the extracellular matrix of *A. fumigatus* (Gravelat et al., 2013; Lee et al., 2015). The bioactive properties of exopolysaccharides are known to depend on many factors, such as the monosaccharide components, molecular mass, conformation, and linkage type (Zhou and Chen, 2011; Elsehemy et al., 2020; Xu et al., 2006). Research on *A. oryzae* has shown that its EPS play a crucial role in supporting microalgal-fungal co-cultivation systems, particularly in wastewater treatment applications. The EPS, consisting of high molecular weight substances including proteins, polysaccharides, and enzymes like amylase, protease, and lipase, contribute significantly to the structural integrity and functionality of these systems (Nie et al., 2022). EPS components facilitate the formation and stabilization of microalgal-fungal aggregates and enhance nutrient removal and biomass production (Wu et al., 2023).

### 5.3 Enzyme production by *Aspergillus oryzae* fermentation

Enzymes are proteins produced by living organisms that serve as catalysts to facilitate specific biochemical reactions (Sundarram and Murthy, 2014; Gurung et al., 2013). Owing to their broad range of applications, the biotechnical production of enzymes through microbial fermentation is an important technology in diverse areas such as production of food, pharmaceuticals, and therapeutics (Cruz-Casas et al., 2021). Fungi are key sources of hydrolytic digestive enzymes, which also play a decisive role in fermentation processes. In the case of *A. oryzae*, several biocatalysts of potential commercial interest have been identified that can be produced via SSF and/or SmF (Table 1). These enzymes, their potential and, in some cases, their current applications are summarized in the following paragraphs.

**Table 1:** Enzymes produced by *Aspergillus oryzae* by solid-state fermentation (SSF) or submerged-fermentation (SmF).

Enzyme	Fermentation method	Fermentation conditions	Substrate	Reference
Amylase				
Amylase	SSF	30°C for 72 h	60% soybean, 40% wheat bran	Chancharoonpong et al. (2012)
α-amylase, glucoamylase	SSF	Optimized parameters: 5.5 pH, 28°C and 72 h, enzyme production was enhanced when ammonium sulfate was added	Wheat bran, sugar beet pulp, sunflower, and corn flour	Fadel et al. (2020)
Glucoamylase	SSF and SmF	SSF: 37°C for 42 h SmF: 30°C for 72 h	SSF: Steamed rice SmF: CZ-Dox medium consisted of 0.3% NaNO_3_, 0.2% KCL, 0.1% KH_2_PO_4_, 0.05% MgSO_4_ x 7H_2_O, 0.002% FeSO_4_ x 5H_2_O, and 3% soluble starch	Hata et al. (1997)
Glucoamylase	SSF	30°C for 5 days	Groundnut oil cake, rice bran, wheat bran, and coconut oil cake	Lakshmi and Jyothi (2014)
Glucoamylase	SSF	30°C for 6 days	Wheat bran + saline containing 0.1% Tween 80	Zambare (2010)
Lipase				
Lipase	SSF	Shaking at 120 rpm at 28°C for 5 days	Rice or soy bean	Ohnishi et al. (1994a)
Lipase	SmF	24°C for 90 h	Culture medium containing 3% soybean oil	Ohnishi et al. (1994b)
Triacylglycerol lipase	SmF	30°C for 4 days	2% peptone, 0.5% yeast extract, 0.1% NaNO_3_, 0.1% KH_2_PO4, 0.05% MgSO_4_ x 7H_2_O, and 2% olive oil (pH 5.5)	Toida et al. (1998)
Lipase	SSF	30°C for 120 h	Palm kernel cake and palm pressed fibre	Oliveira et al. (2018)
Protease				
Protease	SSF	23°C for 72 h	Wheat bran	De Castro and Sato (2014)
Protease	SSF	30°C for 72 h	60% soybean, 40% wheat bran	Chancharoonpong et al. (2012)
Prolyl endopeptidase	SmF	48 h	2% glucose, 1% soy meal protein or 0.2% gliadin, 0.5% KH_2_PO_4_, 0.5% KCL, 0.1% NH_4_CL, 0.05% MgSO_4_, and 68 mM citrate buffer (pH 4)	Eugster et al. (2015)
Neutral protease	SSF and SmF	SSF and SmF: 24, 48, 72, 96, 120, and 144 h at 30°C	SSF substrates: wheat bran, rice husk, rice bran, spent brewing grain, coconut oil cake, palm kernel cake, sesame oil cake, jackfruit seed powder, and olive oil cake in salt solution consisted of 0.1% K_2_HPO_4_, 0.5% MgSO_4_, 0.5% NaCl, and 0.004% FeSO_4_ SmF: 0.1%K_2_HPO_4_, 0.5% MgSO_4_, 0.5% NaCl, and 0.004% FeSO_4_	Sandhya et al. (2005)
Aspergillo-peptidase B	SSF	No data	Wheat bran	Subramanian and Kalnitsky (1964b) and Subramanian and Kalnitsky (1964a)
Alkaline protease	SSF	30°C for 72°C	Wheat bran	Yepuru (2018)
Galactosidase				
α-galactosidase	SSF	37°C for 5 days	1.5% guar gum, 0.6% peptone, 0.15% (NH_4_)_2_SO_4_, 0.6% KH_2_PO_4_ (pH 5.5)	Kulkarni et al. (2006), Prashanth and Mulimani (2005), and Dhananjay and Mulimani (2009)
α-galactosidase	SSF	30°C	Red gram plant waste, red gram flour, red gram husk, pine apple waste, apple waste, orange waste, groundnut cake, sugarcane bagasse, and carob pod + mineral salt solution consisted of 0.63% K_2_HPO_4_, 0.18% KH_2_PO_4_, 0.1% MgSO_4_, 0.01% CaCl_2_, 0.01% FeSO_4_, 0.01% MnSO_4_, and 0.0006% NaMo_7_O_24_	Shankar et al. (2006)
α-galactosidase	SSF	30°C for 4 days	Soda-anthraquinone pulp from *Eucalyptus grandis* and bagasse soda pulp + salt solution consisted of 0.5% NH_4_NO_3_,0.2% corn steep liquor, 0.5% KH_2_PO_4_, 0.1% NaCl, 0.1% MgSO_4_ x 7 H_2_O, 0.0002% CoCl_2_ x 6 H_2_O, 0.0002% MnSO_4_, 0.000345% ZnSO_4_·x 7 H_2_O, and 0.0005% FeSO_4_·x 7 H_2_O (pH 7–9)	Szendefy et al. (2006)
β-galactosidase	SSF and SmF	30°C for 1 week	SSF: wheat bran SmF: 4% soluble potato starch, 1.5% peptone, 1% yeast extract, 1% KH_2_PO_4_, and 0.2% NaNO_3_ (pH 5.5)	Park et al. (1979)
β-galactosidase	SSF	30°C for 6 days	Wheat bran and rice husk	Nizamuddin et al. (2008)
Xylanase				
Xylanase	SSF	30°C for 120 h	Palm kernel cake and palm pressed fibre	Oliveira et al. (2018)
Endo-β-xylanase	SSF	30°C for 4 days	Soda-anthraquinone pulp from *Eucalyptus grandis* and bagasse soda pulp + salt solution consisted of 0.5% NH_4_NO_3_,0.2% corn steep liquor, 0.5% KH_2_PO_4_, 0.1% NaCl, 0.1% MgSO_4_ x 7 H_2_O, 0.0002% CoCl_2_ x 6 H_2_O, 0.0002% MnSO_4_, 0.000345% ZnSO_4_·x 7 H_2_O, and 0.0005% FeSO_4_·x 7 H_2_O (pH 7–9)	Szendefy et al. (2006)
Xylanase	SSF	35°C for 72 h	Wheat bran	Pirota et al. (2013a)
Cellulase				
Cellulase	SSF	Optimized parameters: 5.5 pH, 28°C and 72 h	Wheat bran, sugar beet pulp, sunflower, and corn flour	Fadel et al. (2020)
Cellulase	SmF	35°C for 2–24 h	Vogel’s media: 0.5% Na_3_C_6_H_5_O_7_, 0.5% KH₂PO₄, 0.4% (NH₄)₂SO₄, 0.2% NH₄NO₃, 0.02% MgSO₄, and 0.1% yeast extract (pH 5, 6, and 7) + 1–3% of either wheat, dab grass, and corncobs	Sher et al. (2017)
Cellulase	SSF	30°C for 4 days	Wheat bran + mineral solution consisted of 0.2% KCl, 0.1% KH₂PO₄, and 0.05% MgSO₄·7H₂O (pH 6.0)	Yamane et al. (2002a)
Pectinase				
Pectinase	SmF	25°C to 45°C for 10 days	Pectin broth with 0.5 g to 2.5 g citrus pectin (pH 4 to 9)	Ketipally and Ram (2018)
Pectinase	SmF	28°C for 0 to 168 h	Wheat bran extract liquid medium (8% wheat bran (aqueous extract), 2% citrus pectin, 2.2% glucose, 0.005% yeast extract, 0.5% (NH_4_)_2_SO_4_, 0.05% MgSO_4_, 0.25% KH_2_PO_4_, 0.000063% FeSO_4_·7H_2_O, 0.000062% ZnSO_4_, and 0.000001% MnSO_4_)	Meneghel et al. (2014)

Proteases are enzymes that catalyze the hydrolysis of peptide bonds in proteins, resulting in the formation of smaller peptides or individual amino acids. Their significance in the food industry is multifaceted and has been extensively studied. According to Machida et al. (2005), *A. oryzae* has the largest expansion of hydrolytic genes with 135 proteinase genes including both endo- and exoproteases. Throughout the fermentation process, endoproteases primarily create numerous free termini, facilitating the action of exoproteases (Eugster et al., 2015). In broad bean paste fermentation, the use of a strain of *A. oryzae* with higher protease activity compared to commonly used strains significantly enhances flavor profiles by increasing umami amino acids and volatile flavors, thus improving the sensory characteristics of the final product (Niu et al., 2023). During *A. oryzae* mediated soy sauce fermentation, diverse enzymes are leveraged by the fungus to break down proteins and carbohydrates under high-salt conditions. Early-stage enzymes such as peptidases break down proteins into peptides, while later-stage enzymes like metallopeptidases and extracellular proteinases continue this proteolysis (Zhao et al., 2018). Specifically, leucine aminopeptidase II from *A. oryzae* plays a notable role, accounting for almost 80% of the glutamic acid release from soybean proteins, which essential for the umami flavor of soy sauce (Zhao et al., 2018).

Lipases function as versatile biocatalysts, facilitating a range of reactions such as esterification, hydrolysis, alcoholysis, transesterification, aminolysis, and acidolysis (Zhang et al., 2020). These enzymes have found extensive applications in sectors like the chemical, food, pharmaceutical, and detergent industries (Verma et al., 2012). During the miso fermentation process, lipase from *A. oryzae* facilitates the hydrolysis of glycerides in soybeans. This not only results in the development of distinct flavors and aromas but also leads to notable changes in lipid composition, contributing to the formation of characteristic taste profiles (Ohnishi, 1982).

Esterases are enzymes that break down esters into alcohols and acids. While they share similarities with lipases in breaking down glycerides, esterases specifically target short-chain glycerides (Okpara, 2022). In the beverage industry, feruloyl esterase plays a role in producing ferulic acid. This acid then serves as a starting material for creating the aromatic compound, vanillin, a primary component of vanilla that enhances beverage flavors (Gallage et al., 2014). In brewing sake, *A. oryzae* produces feruloyl esterase, which facilitates the release of ferulic acid from the rice endosperm cell walls. A study examining the effects of this enzyme, specifically the FaeA variant produced by *A. oryzae*, highlighted its significant role in flavor formation (Todokoro et al., 2022).

The enzyme group of amylases, responsible for breaking down starches, consists of two primary categories: amylases and glucoamylase (Lakshmi and Jyothi, 2014). *α*-amylase acts on starch, converting it into maltose, glucose, and maltotriose by targeting the α-1,4-D-glucosidic bonds between glucose molecules in the straight amylase chain. On the other hand, glucoamylase releases individual glucose molecules from the non-reducing ends of both amylose and amylopectin, resulting in the exclusive production of glucose from starches and related compounds (Zong et al., 2022). In a recent study, twenty-five filamentous fungal isolates were tested as potential *α*-amylase sources under SSF conditions using wheat bran as substrate. Among those, an *A. oryzae* isolate (F-923) was the most promising candidate for the production of the target enzyme. Ammonium sulfate supplementation and the addition of soluble starch were found to enhance the α-amylase yield (Fadel et al., 2020).

Galactosidases are a group of enzymes that catalyze the hydrolysis of α- and *β*-galactosidic bonds in certain carbohydrates or glycosides, respectively. These enzymes play a pivotal role in the digestion and assimilation of carbohydrates in various organisms. Among the microorganisms known to produce these enzymes, *A. oryzae* stands out due to its widespread use in industrial applications (Nath et al., 2014). α-galactosidases are specialized in cleaving α-galactosidic bonds, particularly those found in oligosaccharides like raffinose, stachyose, and verbascose. These sugars are commonly found in legumes and certain vegetables and are not digestible by human, which can cause digestive discomfort in some people (Ibrahim et al., 2010). Accordingly, fungal α-galactosidases have the capacity to remove the flatulence-inducing sugars of soymilk and soybean. The results are interesting for their potential use in the food industry (Patil and Mulimani, 2008; Guo YuHan et al., 2018). β-galactosidases target the β-galactosidic bonds, which are present in certain disaccharides like lactose (Saqib et al., 2017). This enzyme is therefore crucial for the digestion of lactose by converting it into glucose and galactose (Swallow, 2003). Lactose intolerance in humans arises from a deficiency of this enzyme. β-galactosidases are frequently used in the dairy industry, especially in the manufacture of lactose-free products (Saqib et al., 2017). The *A. oryzae* β-galactosidase is employed in particular for the production of lactose-free and no-sugar-added yoghurt (Miao et al., 2024). In soy sauce fermentation, β-glucosidase and β-xylanase (see below) are essential for glucose metabolism throughout fermentation, contributing to the development of flavors like alcohols, acids, and esters (Nakadai et al., 1972).

Xylanases are a class of enzymes that degrade the β-1,4-glycosidic linkage between xylose residues of linear polysaccharides (Pirota et al., 2013). As such, they play a crucial role in the decomposition of hemicellulose, one of the major components of plant cell walls (Dodd and Cann, 2009). Given their ability to break down complex carbohydrates, xylanases have garnered significant attention in various industries, particularly in the paper and pulp industry for bleaching paper (Walia et al., 2017) and in the feed industry to improve the digestibility of animal feeds (Ho, 2017; Ojha et al., 2019). Given these applications, *A. oryzae* is used for the production of xylanases due to its efficiency in enzyme synthesis (Pirota et al., 2013; Kimura et al., 2000; Chipeta et al., 2008). Within *A. oryzae*, several specialized xylanases have been identified, including ethanol-tolerant xylanase (Sato et al., 2010), thermo-acid/alkali stable xylanase (Bhardwaj et al., 2019), thermo-alkali-stable xylanase (Bhardwaj et al., 2020), thermostable xylanase (He et al., 2015), and low-molecular-weight xylanase (Duarte et al., 2012). These specialized enzymes highlight the versatility and adaptability of *A. oryzae* in producing xylanases that meet specific industrial needs.

Cellulases are complex enzyme systems responsible for the breakdown of cellulose, the primary structural component of plant cell walls. Cellulose is made up of glucose molecules linked together by β-1,4-glycosidic bonds (Sher et al., 2017). Comprising multiple enzymes, cellulases catalyze the hydrolysis of cellulose into glucose, which can then be utilized by microorganisms or converted into other valuable products. The industrial relevance of cellulases is vast. It plays a pivotal role in several sectors, including the biofuel industry, textile industry, paper and pulp industry, and food industry (Ejaz et al., 2021; Saranraj et al., 2012). In a SSF with *A. oryzae*, four cellulose-degrading enzymes were identified. Notably, one of these identified cellulases significantly enhanced material utilization and alcohol yield during sake mash fermentation (Yamane et al., 2002b).

Pectinases are enzymes that break down pectin and they are categorized based on their action mechanism as polygalacturonases, lyases, and pectin methylesterases (Sudeep et al., 2020). Pectin, a key component in the cell walls of higher plants, consists of high molecular weight acidic heteropolysaccharides primarily made of α-1,4-linked D-galacturonic acid residues (Kavuthodi and Sebastian, 2018). Pectinases, primarily produced through microbes, play a pivotal role in the food and beverage industry, enhancing juice extraction, improving coffee, cocoa, and tea quality, and increasing oil yield in refineries (Sudeep et al., 2020; Tapre and Jain, 2014; Anand et al., 2017). Additionally, they enhance the digestibility of pectin-rich animal feeds (Azzaz et al., 2021; Tahir et al., 2006). *A. oryzae* is often used as the chosen microorganism for pectinase production (Ketipally and Ram, 2018; Meneghel et al., 2014; Chen et al., 2014; Hoa and Hung, 2013; Jaramillo et al., 2015; Hoa and Hung, 2013).

### 5.4 Primary and secondary metabolites produced by *Aspergillus oryzae* fermentation

Numerous analytical studies including targeted and untargeted approaches revealed that *A. oryzae* generate a wide variety of primary and secondary metabolites (Table 2). Typically, primary metabolites are directly associated with an organism’s growth and development and are crucial for its regular physiological activities. Secondary metabolites are not directly linked to normal growth and development, but rather play important roles in virulence, host defense, and environmental survival (Vining, 1990; Pagare et al., 2015; Bennett and Wallsgrove, 1994).

**Table 2:** Metabolites found in *Aspergillus oryzae* fermentation products and their described bioactivity.

Metabolite	Chemical class/family	Occurrence	Bioactivity
Asperorydines A-M	Alkaloid	Fermentation of *A. oryzae* strain L1020 in potato dextrose broth at 25°C for 7 days (Liu et al., 2018a)	Neuroprotective effect (Liu et al., 2018a)
L-ergothioneine	Amino acid derivative	Fermented rice bran with *A. oryzae* (Horie et al., 2020)	Antioxidant activity (Cheah and Halliwell, 2012), cytoprotective effects (Servillo et al., 2017)
Asperfuran	Furan	Fermentation of *A. oryzae* strain HA 302–84 on YMG-medium at 23°C for 48 h (Pfefferle et al., 1990)	Chitin synthase inhibitor (Pfefferle et al., 1990)
Aspirochlorine	Gliotoxin	Fermentation of *A. oryzae* strain IAM 2613 on a medium mainly containing sucrose, peptone, and yeast at 29°C for 20 days (Sakata et al., 1983)	Antibiotic activity (Sakata et al., 1983), antifungal activity (Monti et al., 1999)
Dihydroxymethoxycoumarin	Isocoumarin derivative	Fermentation of *A. oryzae* strain KCCM 12698 on malt extract agar plates or broth at 28°C over 16 days (Son et al., 2018)	Antimicrobial activity (Son et al., 2018)
Ketonecitreoisocoumarin			
Oryzaein A-D		Fermentation of *A. oryzae* isolated from the rhizome of *P. polyphylla* on potato dextrose agar at 28°C for 7 days (Zhou et al., 2016)	Antiviral and cytotoxic activity (Zhou et al., 2016)
Austalide F and H	Meroterpenoid	Fermentation of *A. oryzae* strain KCCM 12698 on malt extract agar plates or broth at 28°C over 16 days (Son et al., 2018)	Austalides in general: Antitumor (Antipova et al., 2019), bacterial inhibitor (Zhou et al., 2014), antiviral (Peng et al., 2016)
9,12,13-TriHODE	Oxylipin	Fermentation of *A. oryzae* strain KCCM 12698 on malt extract agar plates or broth at 28°C over 16 days (Son et al., 2018)	–
9,12,13-TriHOME			–
9,10,13-TriHOME			–
8,11-DiHODE			–
12,13-DiHODE			–
12,13-DiHOME			–
Agmatine	Polyamine	Fermentation of *A. oryzae* strain RW (rice wine) on steamed rice (Akasaka et al., 2018)	Antidepressant-like effect (Zomkowski et al., 2002), anxiolytic activity (Lavinsky et al., 2003), antihyperalgesic effects (Courteix et al., 2007)
Sporogen AO1	Sesquiterpene	Cultivation of *A. oryzae* strain NOY-2 on Czapek-dox dextrose medium in the dark at 30°C for 2 weeks (Tanaka et al., 1984)	Sporulation-stimulating activity (Tamogami et al., 1996), antimalaria activity (Daengrot et al., 2015)
Asperaculin A		Fermentation of *A. oryzae* strain KCCM 12698 on malt extract agar plates or broth at 28°C over 16 days (Son et al., 2018)	–
Heptelidic acid		Fermentation of *A. oryzae* strain ATCC42149 in yeast malt broth for 2–3 days at 37°C (Konishi et al., 2021a)	Anticancer activity (Konishi et al., 2021a), specific inhibitor of glyceraldehyde 3-phosphate dehydrogenase (Endo et al., 1985), antimalarial activity (Tanaka et al., 1998)
Astellolides		Fermentation of *A. oryzae* strain QXPC-4 in liquid Czapek’s medium at 28°C for 12 days (Ren et al., 2015)	Antimicrobial activity (Chen et al., 2022)
Deferriferrichrysin	Siderophore	Fermentation of *A. oryzae* strain F0 on Czapek-Dox minimal medium at 30°C for 64 h (Todokoro et al., 2016)	Iron-chelating activity (Todokoro et al., 2016), anticancer activity

*A. oryzae* has a long history in fermentative metabolite production. A standout feature of *A. oryzae* is the production of kojic acid, a versatile secondary metabolite (Rosfarizan et al., 2010; Yabuta, 1924; Yan et al., 2014) first isolated in 1907 from koji-culture (Bentley, 2006). Kojic acid finds applications as antibiotic (Morton et al., 1945; Rodrigues et al., 2022), food preservative (Wang et al., 2021), and antioxidant (Ermis et al., 2023; Yi and Kim, 1982). Its role as a tyrosinase inhibitor (Saruno et al., 1979) has also propelled its use in cosmetics for skin-lightening (Phasha et al., 2022) and in medicine for chloasma treatments (Monteiro et al., 2013). The biosynthesis pathway of kojic acid in *A. oryzae* is well known (Marui et al., 2011; Terabayashi et al., 2010; Chib et al., 2023).

*Aspergilli* are very suitable for the industrial production of organic acids. Notably, they play a role in the synthesis of the 1,4-dicarboxylic acids like succinic, malic, and fumaric acids. These acids, integral to the tricarboxylic acid (TCA) cycle in all living organisms, have been highlighted by the U.S. Department of Energy as some of the top high-value chemicals derived from biomass (Werpy and Petersen, 2004). While *Aspergillus* species are adept at producing fumaric and malic acids, especially under stress (Shigeo et al., 1962), there are variances in organic acid production capabilities across different strains (Goldberg et al., 2006; Yang et al., 2016). *A. oryzae* is rarely used for the production of succinic acid and fumaric acids, but it has a remarkable potential to produce significant quantities of the former under specific fermentation conditions (Brown et al., 2013; Geyer et al., 2018; Knuf et al., 2013; Knuf et al., 2014; Kövilein et al., 2021; Shigeo et al., 1962). The primary application of malic acid is in the food and beverage sector, where it enhances flavor in products like candies and soft drinks (Kövilein et al., 2020). Its unique taste profile also aids in masking the aftertaste of artificial sweeteners (Aldrich et al., 1979). Beyond this, it finds use in cleaning products (Zhang et al., 2016) and animal feed additive (Stallcup, 1979).

Secondary metabolite production by *Aspergillus* species varies based on fermentation type and conditions (Son et al., 2018). *A. oryzae* produces various secondary metabolites, including terpenoids, coumarins, and oxylipins (summarized in Table 2).

### 5.5 *Aspergillus oryzae* derived postbiotics

**Figure 4:**
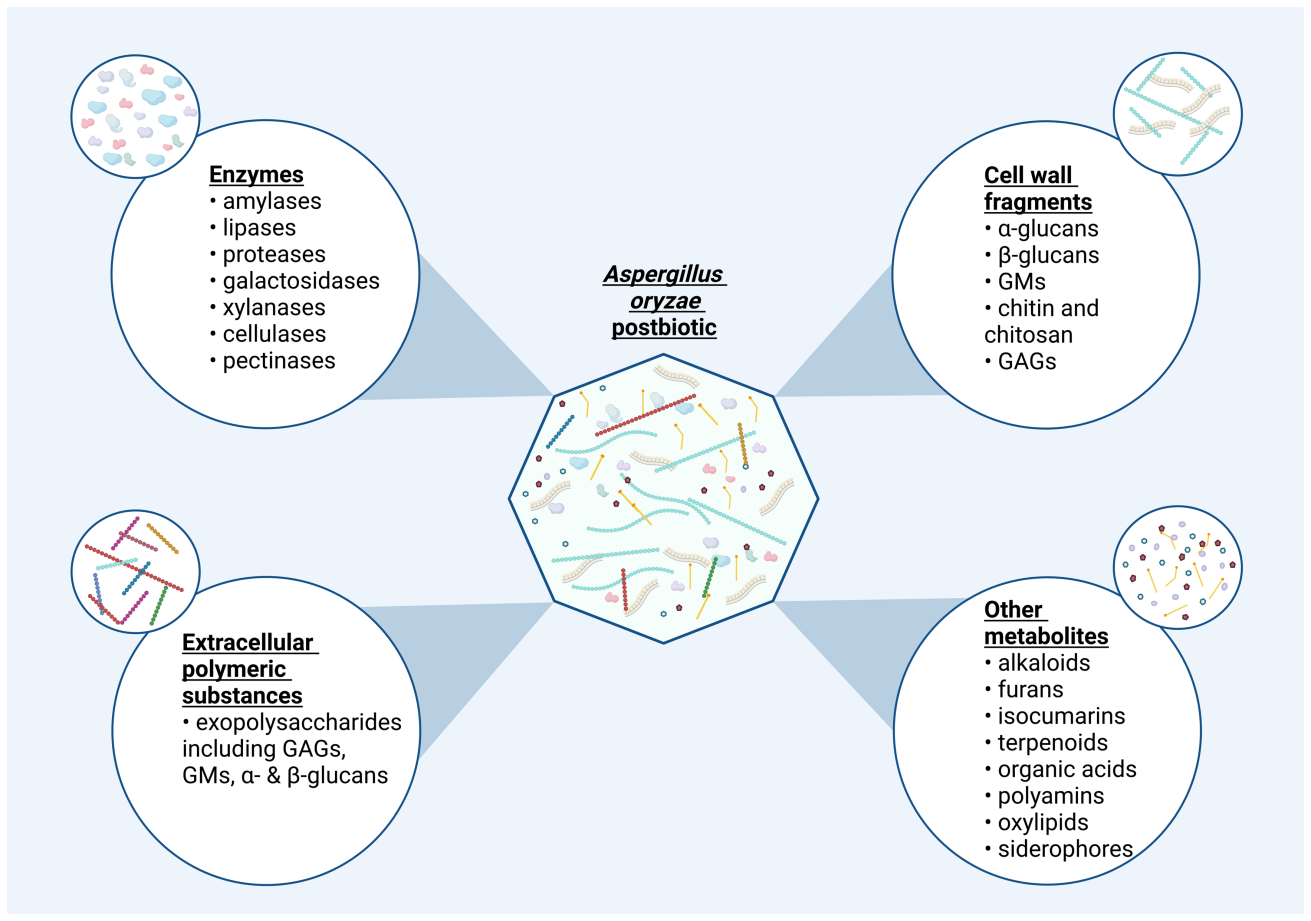
The potential composition of a postbiotic product derived from *Aspergillus oryzae.* Postbiotics produced via fermentation with *A. oryzae* may display a diverse molecular composition. Bioactive compounds in this inanimated fermentation preparations can be organized in four categories: “Non-viable cells or cell-fragments,” “enzymes,” “extracellular polymeric substances,” and “other metabolites”. The latter category is reserved for primary and secondary metabolites not fitting the prior classifications. This categorization provides a structured insight into potential ingredients present in postbiotics derived from *A. oryzae*. Created in BioRender. Seidler, Y. (2024) BioRender.com/f31t998

In the previous sections we have described various compounds derived from the fermentation with *A. oryzae*, the bioactivity of which will next be discussed against the context of their potential use as postbiotics (Figure 4) to confer health benefits on humans or animals. Additionally, it is important to note that the composition and the existing molecules in a potential *A. oryzae* postbiotic are influenced by the inactivation process. Different drying methods can alter the composition of primary and secondary metabolite profile (Managa et al., 2020; Lu et al., 2024; Zagórska et al., 2022), enzymatic activity (Valadez-Carmona et al., 2017; Wang et al., 2018), and the morphology, conformation, and molecular weight of polysaccharides (Kong et al., 2015; Li W. et al., 2021).

## 6 Case studies with *Aspergillus oryzae* postbiotics

The exploration of *A. oryzae* for its health-promoting potential has predominantly been centered around its probiotic (Konishi et al., 2021; Dawood et al., 2020a; Lee et al., 2006; Iwashita et al., 2015) or prebiotic capabilities (Hamajima et al., 2016; Kim et al., 2014; Podversich et al., 2023), which are well-documented in the scientific literature. However, the concept of utilizing *A. oryzae* as a progenitor microorganism for the production of a postbiotic product (or preparation) is a relatively new area of research, as evidenced by the paucity of literature on the postbiotic potential of *A. oryzae*. Certainly, only three published studies have been found (Kaufman et al., 2021; Ríus et al., 2022), which represent pioneering efforts to characterize and harness the postbiotic properties of *A. oryzae*. By employing the fruit fly model *Drosophila melanogaster*, Kaufman et al. (2021) showed that the supplementation of fly diet with an *A. oryzae* postbiotic improved heat stress tolerance. Remarkably, the same *A. oryzae* postbiotic increased milk production and reduced inflammatory markers when fed to dairy cows that were exposed to elevated ambient temperatures. In a follow-up study, Ríus et al. (2022) found that the feeding of *A. oryzae* postbiotic to dairy calves not only mitigated heat-induced reductions in the efficiency of energy use for growth but also improved intestinal roles like barrier function and water absorption, although it did not significantly decrease markers of systemic inflammation. More recently, Junior et al., 2024 examined the effects of *A. oryzae* postbiotics during gestation and lactation in sows, showing a reduction in body weight loss among sows but no significant effect on litter or nursery performance. These findings suggest *A. oryzae* postbiotics could be a valuable tool in improving heat tolerance, physiological functions in various animal species, and potentially reducing weight loss in sows during critical periods.

The term “postbiotic” itself is relatively new to the scientific community, which partly explains the scarcity of studies explicitly addressing *A. oryzae* in this context. Nevertheless, by expanding the search terms to include “inanimate,” “heat killed,” “non-viable,” “inactivated,” “killed,” and “dried,” three additional papers were uncovered (Hymes-Fecht and Casper, 2021; Gomez-Alarcon et al., 1990; Nomura et al., 2022). Although these studies do not label *A. oryzae* explicitly as a postbiotic, they provide valuable insights into its application and potential health benefits in a state that aligns with the broader definition of postbiotics. Investigating the impact on livestock digestive efficiency, Hymes-Fecht and Casper (2021) found that a dried *A. oryzae* fermentation product enhanced the degradation of neutral detergent fiber in selected forages. This led to improvements in nutrient absorption and feed efficiency, suggesting beneficial applications in ruminant diets. In a related study, the effectiveness of dried *A. oryzae* cultures on nutrient utilization was assessed in mature Holstein cows. Their trials showed that dried *A. oryzae* cultures increase the rumen and total tract digestibility of fiber fractions, facilitating better energy extraction from feed and contributing to enhance animal health and productivity (Gomez-Alarcon et al., 1990). Further emphasizing *A. oryzae’s* gastrointestinal benefits, Nomura et al. (2022) demonstrated that heat-killed *A. oryzae* spores significantly increase the population of *Bifidobacterium pseudolongum* in mice. This beneficial anti-inflammatory gut microbe and the alleviation of colitis symptoms underscore *A. oryzae’s* potential to enhance gut health and mitigate inflammatory responses. Overall, these studies (Table 3) can be seen as proof of principle that *A. oryzae* fermentation products can act as postbiotics and help, for instance, to promote digestive health, improve nutrient utilization and increase resistance to inflammation in animals.

**Table 3:** Summary of studies investigating the health and performance benefits of *Aspergillus oryzae* products.

Study	Type of *A. oryzae* product	Organism	Key findings and potential health benefits
Gomez-Alarcon et al. (1990)	Dried *A. oryzae* cultures	Mature Holstein cows	Increased rumen and total tract digestibility of fiber fractions, leading to better energy extraction from feed. This supports the use of *A. oryzae* cultures for enhancing animal health, productivity, and energy utilization.
Kaufman et al. (2021)	Postbiotic (*A. oryzae* fermentation product)	*Drosophila melanogaster* and dairy cows	Improved heat stress tolerance in fruit flies and dairy cows; increased milk production and reduced inflammatory markers in dairy cows exposed to high ambient temperatures. This suggests *A. oryzae* postbiotics could enhance heat tolerance, reduce inflammation, and improve dairy production in animals.
Hymes-Fecht and Casper (2021)	Dried *A. oryzae* fermentation product	Livestock	Enhanced degradation of neutral detergent fiber, leading to improved nutrient absorption and feed efficiency. This demonstrates *A. oryzae’s* potential to improve feed efficiency and nutrient utilization in ruminants.
Ríus et al. (2022)	Postbiotic (*A. oryzae* fermentation product)	Dairy calves	Improved energy use efficiency for growth, enhanced intestinal barrier function and water absorption, but no significant decrease in systemic inflammation markers. These results indicate benefits for digestion, growth, and intestinal health in heat-stressed calves.
Nomura et al. (2022)	Heat-killed *A. oryzae* spores	Mice	Increased population of *Bifidobacterium pseudolongum* and alleviation of colitis symptoms, highlighting *A. oryzae’s* potential for improving gut health and mitigating inflammatory responses.
Junior et al. (2024)	Postbiotic (*A. oryzae* fermentation product)	Sows and piglets	Supplementation with *A. oryzae* postbiotics reduced sow body weight loss during gestation and lactation but had no significant impact on litter or nursery performance. This suggests potential benefits in weight management for sows during critical periods.

## 7 Potential mode of action of *Aspergillus oryzae* postbiotics

The following sections delve into the five key mechanistic components proposed by Salminen et al. (2021) to underpin the beneficial effects of postbiotics. These include modulation of the resident gut microbiota, enhancement of the epithelial barrier function, modulation of local and systemic immune responses, modulation of systemic metabolic responses, and systemic signaling via the nervous system (Salminen et al., 2021). Each mechanism is introduced and explained using examples from organisms other than *A. oryzae*. Subsequently, we proposed associations between these general mechanisms and the specific constituents or molecules found in *A. oryzae.* It is important to note that while we attempted to delineate specific and independent effects, there are considerable overlaps between some mechanistic components that are not easily separable. For example, an influence on the gut microbiota composition can alter the endogenous production of downstream effectors like secondary bile acids (Ridlon et al., 2014) and SCFAs (Silva et al., 2020).

### 7.1 Modulation of the resident gut microbiota

Postbiotics can influence the composition and functionality of the human intestinal microbiome through both direct and indirect means (Ozma et al., 2022). Substances contained in postbiotics, like SCFAs, directly affect the microbiome (Rad et al., 2021). Additionally, EPS from organisms like *Bifidobacterium* interact with the gut flora (Salazar et al., 2008), and certain metabolites like bacteriocins (Huang et al., 2021) and organic acids (Dittoe et al., 2018) can suppress pathogenic activity within the gut.

Additionally, it’s important to note that postbiotic products often include indigestible fibers that modulate the microbiome. This modulation represents a prebiotic-like effect, indicating that the use of postbiotics does not exclude prebiotic benefits. The presence of these indigestible polysaccharides in postbiotics demonstrates their dual functionality, contributing to microbiota modulation through mechanisms typically associated with prebiotics. *A. oryzae* is rich in various polysaccharides, primarily known as indigestible fibers. These compounds, including *β*-glucans (Lam and Chi-Keung Cheung, 2013; Russo et al., 2012), GM (Zartl et al., 2018; Wang H. et al., 2023), chitin and chitosan (Liu et al., 2018b; Guan and Feng, 2022) can act as prebiotics, fostering an environment conducive to the growth of beneficial gut microbiota. Recent research has utilized β-galactosidase from *A. oryzae* to produce novel galactooligosaccharides from lactose. Purified and analyzed oligosaccharides mainly consist of galactose and glucose. Prebiotic activity tests have shown that these oligosaccharides significantly enhance the growth of *Bifidobacterium infantis*, especially at higher concentrations, indicating their potential as prebiotics (Kim et al., 2014). Furthermore, koji contains glycosylceramide, which serves as a prebiotic for *B. coccoides*. Ingestion of purified koji glycosylceramide by mice has led to increased *B. coccoides* abundance in the intestinal microbiota (Hamajima et al., 2016).

### 7.2 Enhancement of epithelial barrier function

The integrity of the gut epithelium is crucial for maintaining overall health, serving as a critical barrier against pathogenic invaders and harmful substances (Celebi Sözener et al., 2020). Strategies to influence the epithelial barrier function include promoting the secretion of proteins such as HM0539 (Gao et al., 2019), reducing inflammation (Schiavi et al., 2016), supporting the functioning of tight junctions (Engevik et al., 2019), and providing protection against LPS-induced disruption (Feng et al., 2018). These effects are well-documented for postbiotics, including preparations from *Lactobacillus plantarum* (Izuddin et al., 2019), *Lactobacillus rhamnosus* GG (Gao et al., 2019), and *Bifidobacterium longum* (Martorell et al., 2021). The use of such postbiotics enhance the integrity and functionality of the epithelial barrier, which is crucial for maintaining gut health and preventing the entry of pathogens.

Enzymes produced during *A. oryzae* fermentation may play a role in this context, potentially influencing the digestibility and bioavailability of nutrients that support epithelial health (Li et al., 2018; He et al., 2020). Xylanase supplementation has been found to improve the intestinal health of broiler chickens, particularly in alleviating barrier impairments caused by *Clostridium perfringens* infections, according to research by Liu et al. (2012). In a separate study, Petry et al. (2020) demonstrated that xylanase also increases gut barrier integrity in growing pigs. The enhancement of the gut barrier can lead to better nutrient absorption and overall improved health outcomes for the pigs, showcasing the broad applicability of xylanase across different species. Further, research has highlighted the role of phytase in the expression of intestinal tight junction and nutrient transporter genes in pigs (Lu et al., 2020). Lastly, a study by Moita et al. (2021) showed that phytase supplementation enhances intestinal health in broiler chickens by potentially modulating the gut microbiota. It promotes the growth of beneficial bacteria and reduces harmful bacteria, which in turn improves intestinal morphology. These changes are associated with increased nutrient digestibility and improved bone parameters, indicating a direct link between enzyme supplementation and enhanced physiological development in poultry. Additionally, potential constituents of postbiotics from *A. oryzae*, such as certain furans and alkaloids, may possess the ability to impact directly tight junction proteins (Guo et al., 2020). Furthermore, the expected prebiotic-like effect of preparations from *A. oryzae* could also influence epithelial barrier function through alterations in the gut microbiome (Rose et al., 2021; Camilleri, 2021).

### 7.3 Modulation of local and systemic immune responses

**Figure 5:**
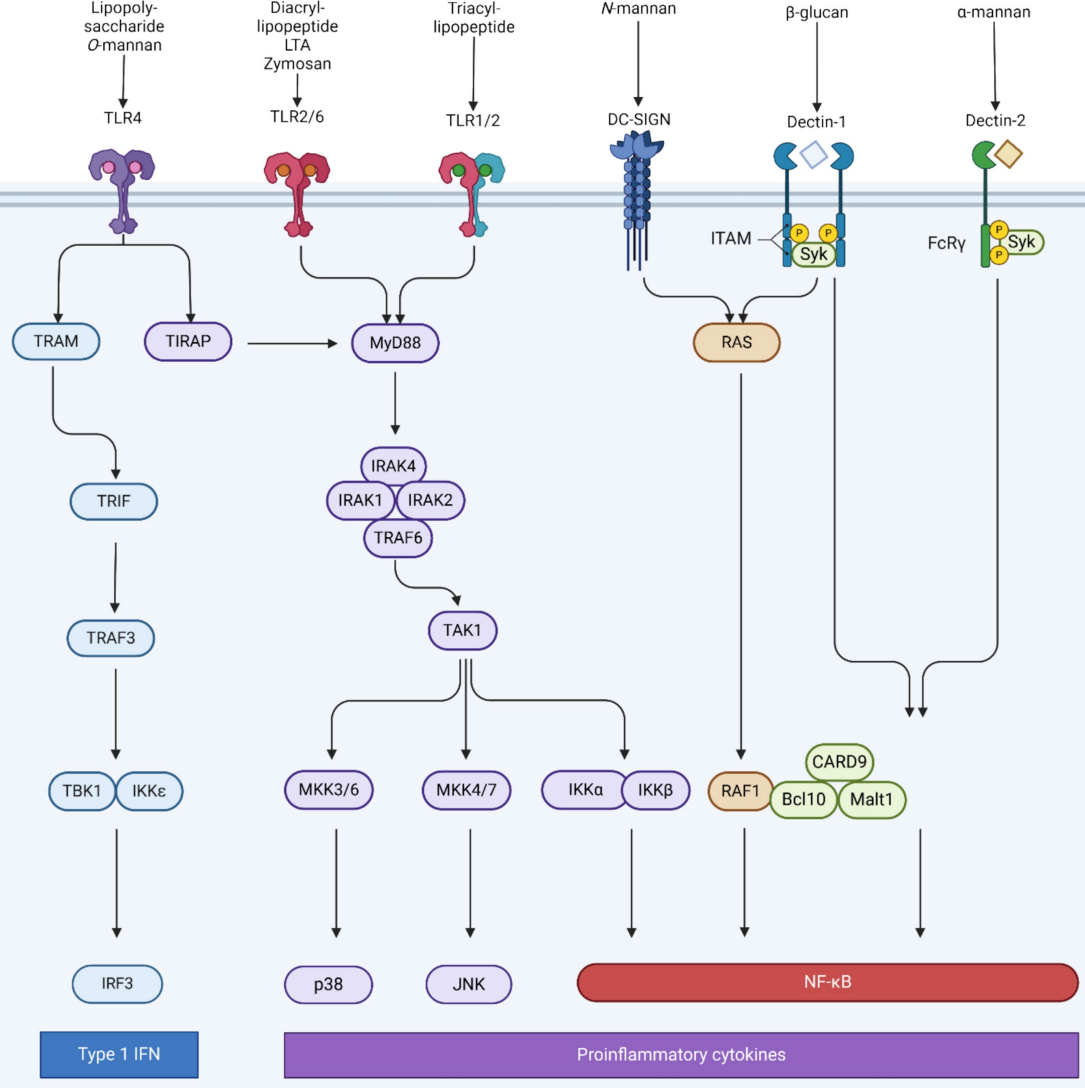
Receptors involved in antifungal immunity, their intracellular signaling pathways and their corresponding fungal PAMPs. Recognition of fungi cell wall components of the cell membrane is mainly mediated by toll-like receptors (TLRs) and C-type lectin receptor (CLRs). TLR2 and TLR4 both signal to interleukin-1 receptor-associated kinase (IRAKs) through myeloid differentiation primary response 88 (MyD88). An IRAK4/IRAK1/IRAK2/tumor necrosis factor receptor-associated factor 6 (TRAF6) complex activates transforming growth factor-beta activated kinase 1 (TAK1) which leads to the activation of the IkappaB kinase (IKK) complex and the mitogen activated protein (MAP) kinase cascade (mitogen activated protein kinase kinase (MKK) 3/6 and MKK4/7), which finally leads to the nuclear translocation of pro-inflammatory transcription factor nuclear factor-kappa B (NF-κB), activator protein (AP-1), and interferons regulatory factor 3 (IRF3). Each transcription factor is responsible for the transcription of specific genes that encodes different set or proteins such as pro-inflammatory cytokines. TLR4 activation also triggers the secretion of interferon α (IFN-α) and IFN-β promoted by Toll/IL-1R domain-containing adapter-inducing factor (TRIF) -mediated interferons regulatory factor 3 (IRF3). Dectin-1 and dendritic cell-specific intercellular adhesion molecule-3-grabbing non-integrin (DC-SIGN) also activate NF-κB signaling via the rat sarcoma-rapidly accelerated fibrosarcoma 1 (RAS–RAF-1) pathway. Moreover, dectin-1 and dectin-2 recruit the spleen tyrosine kinase (Syk) to form a caspase recruitment domain containing protein 9 (CARD9)/ B-cell lymphoma 10 (BCL-10) / mucosa-associated lymphoid tissue lymphoma translocation protein 1. (MALT1) complex, which activates NF-κB. FcγR, Fcγ receptor; IL, interleukin; JNK, Jun N-terminal kinase; ITAM, immunoreceptor tyrosine-based activation motif. Created with Biorender.com.

Postbiotics are primarily associated with immunoregulatory functions, as they activate both the adaptive and innate immune responses, maintain the integrity of the intestinal mucosal barrier, and counteract microorganisms through the production of antibiotic substances (Ozma et al., 2022). Building upon the comprehensive understanding of the *Aspergillus* species’ cell wall intricacies, it is imperative to explore the broader biological implications of these molecular constituents. The fungal cell wall, abundant with diverse polysaccharides and biomolecules, serves as the primary interface between the fungus and its host, orchestrating a myriad of immunological interactions. These components, recognized as pathogen-associated molecular patterns (PAMPs), are adeptly discerned by the host’s immune surveillance mechanisms. This recognition is facilitated by a sophisticated network of immune cell receptors, notably the Toll-like receptors (TLRs) and C-type lectin receptors (CLRs). Tailored to detect specific fungal PAMPs, these receptors initiate a series of immune responses aimed at countering the fungal intrusion (see Figure 5).

Central to the recognition of fungi by the immune system are pattern recognition receptors (PRRs) that identify fungal PAMPs (Amarante-Mendes et al., 2018). There are four major sub-families of PRRs, namely (i) the TLRs, (ii) the nucleotide-binding oligomerization domain (NOD)- like receptors (NLR), (iii) the retinoic acid-inducible gene (RIG)-like receptors, and (iv) the CLRs (Walsh et al., 2013). Among the PRRs, TLRs play a pivotal role in fungal recognition. For instance, TLR2 and TLR4 recognize fungal cell wall components like phospholipomannan and *O*-linked mannan, respectively (Netea et al., 2008). However, the recognition of fungi is not limited to TLRs. CLRs such as Dectin-1 and Dectin-2 receptor are paramount in sensing *β*-glucans and the macrophage mannose receptor (MR) recognized *N*-linked mannans in the fungal cell wall (Netea et al., 2008). NLRs and RIG-like receptors are another layer to the immune surveillance against bacteria. So far, no studies have documented the involvement of NLRs and RIG-I-like receptors in the recognition of fungi (Netea et al., 2008). It is known that the addition of *A. oryzae* to primary human corneal epithelial cells (HCEC) leads to the upregulation of TLR2 and TLR4 (Ballal et al., 2017). Similarly, in male broiler chicks, the mRNA expression of immune mediators in the intestine was studied, showing that TLR4 expression in the *A. oryzae* and antibiotic groups was higher than in the control group (Takahashi, 2012).

In many biological scenarios, the activation of cellular receptors is not confined to a singular entity; rather, multiple receptors can be concurrently activated. It is important to acknowledge that PRRs can exhibit inhibitory interactions, particularly when faced with a variety of pathogens. Ferwerda et al. (2008) showed that Dectin-1 receptor has potent synergistic effects with both TLR2 and TLR4 in human peripheral blood mononuclear cells (PBMCs) and macrophages. Loures et al. (2015) indicated that TLRs and CLRs are both involved in the induction of lymphocyte proliferation and Th17/Tc17 differentiation mediated by *Paracoccidiodes brasiliensis* activated dendritic cells (DCs), but a synergist action was restricted to Dectin-1 receptor, TLR-4, and MR. Other research showed that pure TLR2 and TLR4 ligands generate macrophages with a diminished ability to produce inflammatory cytokines. In contrast, mouse hematopoietic stem and progenitor cells (HSPCs) activation in response to *Candida albicans* leads to the generation of macrophages that are better prepared to deal with the infection, as they produce higher amounts of inflammatory cytokines and have higher fungicidal capacity than control macrophages (Megías et al., 2016). It was reported that simultaneous stimulation with TLR2 and TLR4 ligands results in the production of tumor necrosis factor alpha (TNF-*α*) at levels much greater than that observed for each of the ligands alone (Sato et al., 2000). Research has also identified other combinations of TLR ligands in DCs that can boost the production of IL-12 and IL-23, leading to DCs with enhanced and sustained T helper type 1–polarizing capacity (Napolitani et al., 2005). This combined TLR stimulation has been used to create an adjuvant to improve T cell responses to vaccines. For instance, stimulating with three TLRs (TLR2/6, TLR3, and TLR9) can not only increase the number of T cells but also change the quality of the immune response, promoting the growth of regulatory T cells (Zhu et al., 2010). In summary, the combined stimulation of TLRs and CLRs, especially Dectin-1 receptor, significantly influences immune responses. The distinct reactions of macrophages and DCs highlight their unique roles in immunity. The synergistic effects of specific combinations, particularly in vaccine adjuvants, open promising therapeutic avenues, emphasizing the potential of targeted immune modulation in addressing infections.

In *Nile tilapia*, various studies have demonstrated that the use of *A. oryzae* as probiotic positively impacts the immune system, oxidative stress response, growth, and disease resistance (Dawood et al., 2020b; Dawood et al., 2020a; Iwashita et al., 2015). In red sea breams fed with *A. oryzae* fermented rapeseed meal, a significantly enhanced immunological response was observed (Dossou et al., 2018). In a study in which mice underwent acute inflammation caused by ear edema, the feeding of a diet containing 10% rice bran with *A. oryzae* reduced inflammation severity (Umeyama et al., 2021). Similarly, it has been shown in cattle that supplementation with an *A. oryzae* postbiotic reduced inflammation in response to heat stress (Kaufman et al., 2021). In a recent *in vitro* study, *A. oryzae* fermentation extract inhibited *Mycoplasma pneumoniae* growth and invasion into A549 lung epithelial cells and reduced the production of TNF-α and IL-6 in murine MH-S alveolar macrophages. Subsequently, in an *in vivo* mouse model of pneumonia, the extract decreased neutrophil infiltration and lung inflammation, demonstrating its potential as a therapeutic agent (Lee et al., 2023).

In summary, the comprehensive understanding of *Aspergillus* species’ cell wall components and their interactions with the host’s immune system highlights the potential for *A. oryzae*-derived postbiotics to exert significant immunomodulatory effects. These postbiotics may have the capability to modulate both local and systemic immune responses, thereby potentially enhancing host defense mechanisms against pathogens.

### 7.4 Modulation of systemic metabolic responses

The influence on systemic metabolic responses could stem directly from the metabolites or enzymes harbored within inactive microorganisms commonly found in postbiotics (Salminen et al., 2021). For instance, a recent study conducted by Travers et al. (2016) showcased that the supernatant of *Lactobacillus johnsonii*, regarded as a postbiotic, harbors active elements proficient in transforming benign bile constituents into markedly toxic compounds for *Giardia duodenalis*. This exemplifies how postbiotics can influence bile acid metabolism, underscoring their potential in addressing parasitic infections. Furthermore, *A. oryzae* fermentation end products have been implicated in influencing bile acids. Feeding obese mice koji glycosylceramide led to a significant reduction in liver cholesterol levels, likely due to cholesterol conversion into bile acids. This indicates that koji glycosylceramide impacts both bile acid and cholesterol metabolism in obese mice (Hamajima et al., 2019). The secondary metabolites produced by *A. oryzae* have been shown to possess various biological properties, including antioxidative, antimicrobial, and antitumor effects (see Table 2). These properties suggest a potential role for *A. oryzae* postbiotics in modulating systemic metabolic responses, although the specific mechanisms through which these effects are mediated remain to be fully elucidated. The concentration and bioavailability of these metabolites in the final product are likely crucial factors dictating their biological efficacy.

### 7.5 Systemic signaling via the nervous system

By signaling through the nervous system and ultimately altering the delivery of neuroactive compounds, microorganisms can influence host behavior and cognitive function. This interplay, enriched by microbiome biotransformation, exposes the host to bioactive products affecting gastrointestinal-central nervous system communication. These compounds modulate central physiological and pathological processes via receptor binding, vagus nerve stimulation, neurotransmission, and neuroinflammation. Therefore, understanding the impact of SCFA, bile acids, neurotransmitters, and other microbial products in the gut-brain axis is crucial (Caspani and Swann, 2019).

While direct evidence linking *A. oryzae* postbiotics to systemic signaling via the nervous system is limited, the potential for β-glucans (Singh and Bhardwaj, 2023), glucosylceramides (Hamajima et al., 2019), and other polysaccharides (Nomura et al., 2022) to impact the gut-brain axis is an area of great interest. These components could modulate neurological health and behavior through their effects on the gut microbiota, bile acid metabolism, and immune function, highlighting an exciting frontier for future research.

## 8 Challenges and opportunities in quality control of fungal postbiotics

Within the realm of postbiotic research, the advent of fungal-derived postbiotics represents a step toward exploring a previously untapped source of beneficial compounds for human and animal health. The process of inactivating microbial cells to produce postbiotics poses no greater challenge for fungi than it does for bacteria.

Techniques such as spray drying (Stephan and Zimmermann, 1998), high-pressure processing (Pinto et al., 2020), heat inactivation (Silva, 2020), thermosonication (Silva, 2020), and the creation of lysates are equally applicable to fungal cells, providing a robust framework for the development of fungal postbiotics. However, the rigorous quality control and quantification of these fungal postbiotics emerge as a paramount challenge, underscored by the unique biological characteristics of fungi and the complex biochemical composition of their derivatives.

In their publication, Salminen et al. (2021) recognized the necessity of providing clear guidance on the technical aspects of postbiotic characterization and quantification. For bacteria, metrics such as colony-forming units (CFU) and cell sorting techniques offer straightforward quantification methods. In contrast, the fungal domain presents a more intricate scenario. Fungal cells, with their varied sizes, multicellular structures, and resilient spore forms, necessitate the development of bespoke quantification strategies that go beyond the bacterial paradigms. It is noteworthy that in most studies focusing on koji production, the quantification of GlcNAc has been utilized as an index for the mycelial weight of *A. oryzae* (Ferdouse et al., 2019). In the study by Ferdouse et al. (2019), alongside the quantification of GlcNAc, the content of glycosylceramide was measured.

Current methodologies, including flow cytometry, can adeptly measure inanimate intact microbes, distinguishing between live, dead, and damaged cells (Robertson et al., 2021; Duquenoy et al., 2020; Baymiev et al., 2020). However, analyzing fungi is, often requiring indirect quantification like biomass estimation, which may not fully capture the postbiotic potential. Flow cytometry is underutilized for filamentous fungi due to the size of hyphae, which cannot pass through the system, limiting analysis to early-stage spores (Mathis et al., 2020). Despite this, it shows promise for spore sorting and viability testing. For fungi like Trichoderma, conidia can be effectively analyzed using various dyes and markers like GFP, with protocols for preparing and staining cells (Steiger, 2021).

Moreover, the identification and quantification of metabolites, which is crucial for understanding the bioactive profile of fungal postbiotics, may demand the use of sophisticated analytical techniques such as high-performance liquid chromatography (HPLC) and mass spectrometry. Yet, the heterogeneity of fungal metabolites and the need for specific markers for bioactivity assessment pose significant hurdles in standardizing these measures.

## 9 Conclusion and future perspectives

Although *A. oryzae* has long been used in the food sector, its application as a postbiotic is still in its infancy. An important basis for assessing its postbiotic potential is the systematic analysis of its components, which has only recently been initiated. As summarized here, numerous interesting molecules have already been identified, including secondary metabolites, cell wall components and enzymes, whose different bioactivities could potentially cover all five of the postbiotic targets described by Salminen et al. (2021), suggesting a broad spectrum of health benefits. Nevertheless, these analyses have not yet been completed. For example, a detailed understanding of the structure of the cell wall is still lacking. It can also be assumed that further bioactive substances produced by *A. oryzae* will be discovered in the future. Most of all, however, there is currently a lack of controlled studies investigating the postbiotic potential of *A. oryzae in vivo*. The limited published research primarily focused on the impacts of *A. oryzae* postbiotics on animal health and nutrition. Findings from those studies suggest that *A. oryzae* fermentation end products, whether in dried or otherwise inactivated form, may confer beneficial effects by improving gut health, promoting immune responses, and enhancing nutrient absorption. Hence, we conclude that the exploration of *A. oryzae* as a source of postbiotics represents a promising avenue in the field of biotics. However, to unlock the full therapeutic potential of *A. oryzae*-derived postbiotics and deepen our understanding of their role in promoting health and well-being, future research should aim to elucidate the specific mechanisms of action of *A. oryzae* postbiotics and investigate their therapeutic applications in different health and disease contexts.

As postbiotics from fungi such as *A. oryzae* is a relatively new research field, it also presents challenges in manufacturing and quality control. Standardized fermentation protocols are required to guarantee reproducible compositions of postbiotic products. Moreover, while the methods for inactivating fungi are broadly similar to those for the much better established bacterial postbiotics, a major challenge is the development of rigorous quality assurance and quantification methods of these novel fungal products. Meeting this demand will require a concerted effort by researchers utilizing state-of-the-art technologies and interdisciplinary approaches to create robust standards for the development of fungal postbiotics. Indeed, commitment in this area will be crucial to realize the full potential of fungal postbiotics for supporting human and animal health.
